# Data on banana transcriptome in response to blood disease infection

**DOI:** 10.1016/j.dib.2020.105133

**Published:** 2020-01-29

**Authors:** Fenny Martha Dwivany, Husna Nugrahapraja, Aisha Rizky Rahmawati, Dwianty Putri Meitasari, Audie Masola Putra, Popi Septiani, Nadya Farah, Siti Subandiyah

**Affiliations:** a School of Life Sciences and Technology, Institut Teknologi Bandung, Bandung 40132, Indonesia; b Biosciences and Biotechnology Research Center, Institut Teknologi Bandung, Bandung 40132, Indonesia; c Bali International Research Center for Banana, Badung-Bali 80361, Indonesia; d Faculty of Agriculture, Gadjah Mada University, Yogyakarta 5528, Indonesia; e Research Centre for Biotechnology, Gadjah Mada University, Yogyakarta 55281, Indonesia

**Keywords:** Defense mechanism, Disease resistance, Gene expression, Pathogen, RNA-seq

## Abstract

Blood disease of Banana (BDB) is one of the prevalent disease caused by *Ralstonia syzygii* subsp. *celebesensis* (Rsc) which cause substantial loss on banana production in Indonesia. To date, the genetic basis of plant defense mechanism caused by blood disease in banana is not available. As a matter of fact, the knowledge of global gene expression will provide important information on plant response to the pathogen infection. Data from transcriptomic analysis in response to blood disease infection from *Musa acuminata* cv. Mas Kirana (AA group), representing the A genome, and *Musa balbisiana* cv. Klutuk (BB group), representing the B genome, were firstly reported. The transcriptome data discussed in this publication are accessible through NCBI's Gene Expression Omnibus with GEO Series accession number GSE138749. These data provide the basis for further investigation on the global gene expression which is pivotal to understand the mechanism of disease resistance from two banana genomes in response to blood disease infection.

**Table:** Specification Table

Subject Area	Host plant-pathogen interaction
More specific subject area	Transcriptomics
Type of data	Abundance measurements generated from RNA sequencing data
How data were acquired	Illumina HiSeq 2500
Data format	Raw (FASTQ) sequences and Tab-delimited text files with FPKM values
Experimental factors	RNA sequencing was performed by using Illumina Hiseq 2500
Experimental features	RNA sequencing from banana pseudo stem of un-inoculated and Rsc inoculated condition
Data source location	Bandung, West Java, Indonesia (6°53′28.9″S 107°36′38.3″E)
Data accessibility	The data discussed in this publication were deposited in NCBI's Gene Expression Omnibus [3] and are accessible through GEO Series accession number GSE138749 https://www.ncbi.nlm.nih.gov/geo/query/acc.cgi?acc=GSE138749

**Table:** 

**Value of the Data** • These data provide the first information of banana transcriptome from two different genomes (A and B) in response to blood disease infection caused by *Ralstonia syzgii* subsp. *celebesensis* (Rsc). • These data will be important to investigate the global gene expression in two different banana. Genomes for identifying candidate genes involved in the banana response to Rsc infection. • These data will be useful for genetic improvement of banana for resistance to blood disease.

## 1 Data description

These data provide transcriptomics approaches to contribute to the understanding defense mechanism of blood disease (*Ralstonia syzgii* subsp. *celebesensis*; Rsc) resistance in A and B banana genome. The raw data include information about RNA-sequencing statistics, transcript mapping and identification. All the transcriptomics data were deposited in NCBI's Gene Expression Omnibus [3] and are accessible through GEO Series accession number GSE138749 (https://www.ncbi.nlm.nih.gov/geo/query/acc.cgi?acc=GSE138749). All samples of RNA-seq data are available at NCBI's Sequence Read Archive (SRA) database with the accession number SRP225171. Some transcriptomic dataset statistics are provided in Table 1, Table 2.

**Table 1:** Output statistics of RNA-sequencing from un-inoculated (C) and Rsc inoculated (I) of *Musa acuminata* cv. Mas Kirana (MA) and *Musa balbisiana* cv. Klutuk (MB) in each replicate (R) of paired-end experiment.

No	Samples ID*	Total Reads	GC percentage
1	MA_C_R1_1.fastq.gz	19,985,442	48
2	MA_C_R1_2.fastq.gz	19,985,442	48
3	MA_C_R2_1.fastq.gz	21,411,565	48
4	MA_C_R2_2.fastq.gz	21,411,565	48
5	MA_C_R3_1.fastq.gz	18,656,400	48
6	MA_C_R3_2.fastq.gz	18,656,400	48
7	MA_I_R1_1.fastq.gz	22,371,766	49
8	MA_I_R1_2.fastq.gz	22,371,766	49
9	MA_I_R2_1.fastq.gz	24,663,768	52
10	MA_I_R2_2.fastq.gz	24,663,768	52
11	MB_C_R1_1.fastq.gz	17,769,678	51
12	MB_C_R1_2.fastq.gz	17,769,678	51
13	MB_C_R2_1.fastq.gz	19,287,762	50
14	MB_C_R2_2.fastq.gz	19,287,762	50
15	MB_C_R3_1.fastq.gz	20,142,652	52
16	MB_C_R3_2.fastq.gz	20,142,652	52
17	MB_I_R1_1.fastq.gz	19,444,312	50
18	MB_I_R1_2.fastq.gz	19,444,312	50
19	MB_I_R2_1.fastq.gz	22,113,556	50
20	MB_I_R2_2.fastq.gz	22,113,556	50

*All samples from RNA-sequencing data are available at NCBI's Sequence Read Archive (SRA) database with the accession number: SRP225171.

**Table 2:** Mapping results and transcripts detection from un-inoculated (C) and Rsc inoculated (I) in each replicate (Rep) of *Musa acuminata* cv. Mas Kirana (AA group) (MA) and *Musa balbisiana* cv. Klutuk (BB group) (MB).

No.		Sample	Mapped Reads (%)	Transcripts Detected	Accession number
1		MA_C_Rep1.txt	77	28,809	GSM4118638
2		MA_C_Rep2.txt	78.3	29,411	GSM4118639
3		MA_C_Rep3.txt	76.8	27,398	GSM4118640
4		MA_I_Rep1.txt	82.1	28,605	GSM4118641
5		MA_I_Rep2.txt	86.7	24,992	GSM4118642
6		MB_C_Rep1.txt	86.8	21,426	GSM4118643
7		MB_C_Rep2.txt	85.1	24,766	GSM4118644
8		MB_C_Rep3.txt	84.7	24,697	GSM4118645
9		MB_I_Rep1.txt	84.4	25,232	GSM4118646
10		MB_I_Rep2.txt	85.6	25,707	GSM4118647

## 2 Experimental design, materials and methods

The Rsc strain was isolated from blood diseases-infected banana which were grown in West Java, Indonesia. In this study, *Musa acuminata* cv. Mas Kirana (AA group), representing the A genome, and *Musa balbisiana* cv. Klutuk (BB group), representing the B genome, were inoculated with 2.5 ml of 10^7^ cfu/ml Rsc suspension using injection method into pseudo stem tissue. The total RNA was extracted from pseudo stem tissue from each banana genotype in un-inoculated condition as control (triplicates) and inoculated condition (duplicates) on seventh days after Rsc inoculation then extracted using Cordeiro method [1].

The quality and concentration of RNA were examined with 1% agarose gel electrophoresis and NanoDrop spectrophotometer (NanoDrop Technologies, USA) at 230, 260, and 280 nm. The cDNA synthesis was performed using the iScript™ cDNA Synthesis kit in 96-Well Fast Thermal Cycler (Thermo Fisher Scientific Inc., USA). The RNA library from Mas Kirana (AA group) and Klutuk (BB group) were constructed using TruSeq RNA Sample Prep KIT v2 and were sequenced using Ilumina platform Hiseq 2500. The quality control of each sample was conducted to examine possible low base score, Illumina adapter and PCR contaminations using FastQC program [4]. The software Cutadapt was used to remove adapter sequences and PCR contaminations in order to get clean reads [5]. The clean reads were mapped to *Musa acuminata* DH Pahang V2 (https://banana-genome-hub.southgreen.fr/sites/banana-genome-hub.southgreen.fr/files/data/fasta/version2/musa_acuminata_v2_pseudochromosome.fna) [6] and *Musa balbisiana* PKW V1 (https://banana-genome-hub.southgreen.fr/sites/banana-genome-hub.southgreen.fr/files/data/fasta/pkw/PKW_pseudochromosome.fa) [2] genome references using TopHat-Cufflink pipeline [7]. Reads that aligned to annotated loci were quantified and normalized with FPKM value using Cufflinks V2.2.1 [8].

## Author contributions

Fenny Martha Dwivany: Conceptualization, Methodology, Validation, Writing-Original Draft preparation Husna Nugrahapraja: Resources, Data Curation, Validation, Methodology Aisha Rizky Rahmawati: Formal Analysis, Investigation, Writing-Original Draft Dwianty Putri Meitasari: Investigation, Formal Analysis, Writing Audie Masola Putra: Writing-Review and Editing Popi Septiani: Formal Analysis, Writing-Original Draft Preparation Nadya Farah: Investigation, Writing-Original Draft Preparation Siti Subandiyah: Writing-Reviewing and Editing, Supervision.
